# Cell Type-Specific Structural Organization of the Six Layers in Rat Barrel Cortex

**DOI:** 10.3389/fnana.2017.00091

**Published:** 2017-10-13

**Authors:** Rajeevan T. Narayanan, Daniel Udvary, Marcel Oberlaender

**Affiliations:** Max Planck Group: In Silico Brain Sciences, Center of Advanced European Studies and Research, Bonn, Germany

**Keywords:** barrel cortex, whisker touch, soma, dendrite, axon

## Abstract

The cytoarchitectonic subdivision of the neocortex into six layers is often used to describe the organization of the cortical circuitry, sensory-evoked signal flow or cortical functions. However, each layer comprises neuronal cell types that have different genetic, functional and/or structural properties. Here, we reanalyze structural data from some of our recent work in the posterior-medial barrel-subfield of the vibrissal part of rat primary somatosensory cortex (vS1). We quantify the degree to which somata, dendrites and axons of the 10 major excitatory cell types of the cortex are distributed with respect to the cytoarchitectonic organization of vS1. We show that within each layer, somata of multiple cell types intermingle, but that each cell type displays dendrite and axon distributions that are aligned to specific cytoarchitectonic landmarks. The resultant quantification of the structural composition of each layer in terms of the cell type-specific number of somata, dendritic and axonal path lengths will aid future studies to bridge between layer- and cell type-specific analyses.

More than a century ago, Brodmann (Brodmann, 1909) described that the shapes and diameters of neuron somata vary as a function of cortical depth (see Garey, 1994 for an English translation of Brodmann's original work). These differences correlate with systematic changes in neuron densities along the vertical cortex axis (i.e., from the pial surface toward the white matter), which gave rise to the concept of cytoarchitectonic layers (Brodmann, 1909). The neocortex is typically subdivided into six layers, i.e., layers 1-6 (L1-6). Even though cortical layers are purely defined by vertical gradients in soma size, shape or density, and there are many exceptions to the division into six layers across cortical areas and species (see Elston, 2002, 2003; Spruston, 2008; DeFelipe, 2011; Elston et al., 2011; Kaas, 2013; Rockland, 2017, for comparative studies and reviews on some aspects of structural cortical heterogeneity), this concept has been widely accepted to provide a first order criterion to discriminate between neuronal cell types. Countless studies have thus grouped neurons by their laminar soma locations and provided layer-specific analyses.

Moreover, layers are often used as a synonym for elementary computational units when describing the organization of cortical circuits. For example, in primary sensory cortices, a general motif, referred to as a “canonical circuit” has been proposed (reviewed in Douglas and Martin, 2004). According to this theory, L4 is regarded as the major thalamorecipient layer and thus as the starting point of cortical information processing. Information from L4 is then thought to propagate through the cortical column, first to L2/3 from where it is then relayed to L5/6, the primary output layers of the cortex. However, each layer is populated by genetically (e.g., Zeisel et al., 2015; Tasic et al., 2016), biophysically (e.g., Ferrante et al., 2017), physiologically (e.g., de Kock et al., 2007) and morphologically (e.g., Narayanan et al., 2015) diverse neuron populations. This heterogeneity of each layer—as well as the general structural heterogeneity across cortical areas and species—raises the question to what extent grouping of neurons by their laminar soma locations is an appropriate simplification to study and describe the structural and functional organization of cortical circuits.

In this review article, we reanalyze some of our recent work about the structural and functional organization of the posterior-medial barrel-subfield in the vibrissal part of rat primary somatosensory cortex (vS1, i.e., barrel cortex). On the example of this well-studied, yet—when compared to other cortical areas and species—uniquely organized primary sensory area (e.g., the rodent barrel cortex is characterized by neuron-dense aggregates in L4 (i.e., barrels) that form a somatotopic map of the facial whiskers), we seek to address the following general questions: *How layer-specific are physiological and morphological properties of individual excitatory neurons, and how homogeneous are cortical layers with respect to the resultant structure-function neuronal cell types?*

First, we define the borders between the six layers of rat vS1 by precise measurements of the 3D distributions of all excitatory and inhibitory neuron somata (Meyer et al., 2013). Second, we group *in vivo* recorded excitatory neurons, whose dendrite and axon morphologies have been reconstructed, into 10 structure-function cell types (Oberlaender et al., 2012; Narayanan et al., 2015). Third, these datasets are combined by registration into a precise anatomical reference frame of rat vS1 (Egger et al., 2012), which allows quantifying cell type-specific soma, dendrite and axon distributions with respect to the six layers of vS1. Finally, we determine the number of somata, as well as the amounts of dendrites and axons that each of the ten cell types contributes to each of the six layers within the volume of an average cortical barrel column. The present review article thus provides a quantitative account of the structural heterogeneity of cortical layers, which will help relating layer-specific to cell type-specific studies of cortex organization.

## Defining cortical layers in rat vS1

**Figure 1:**
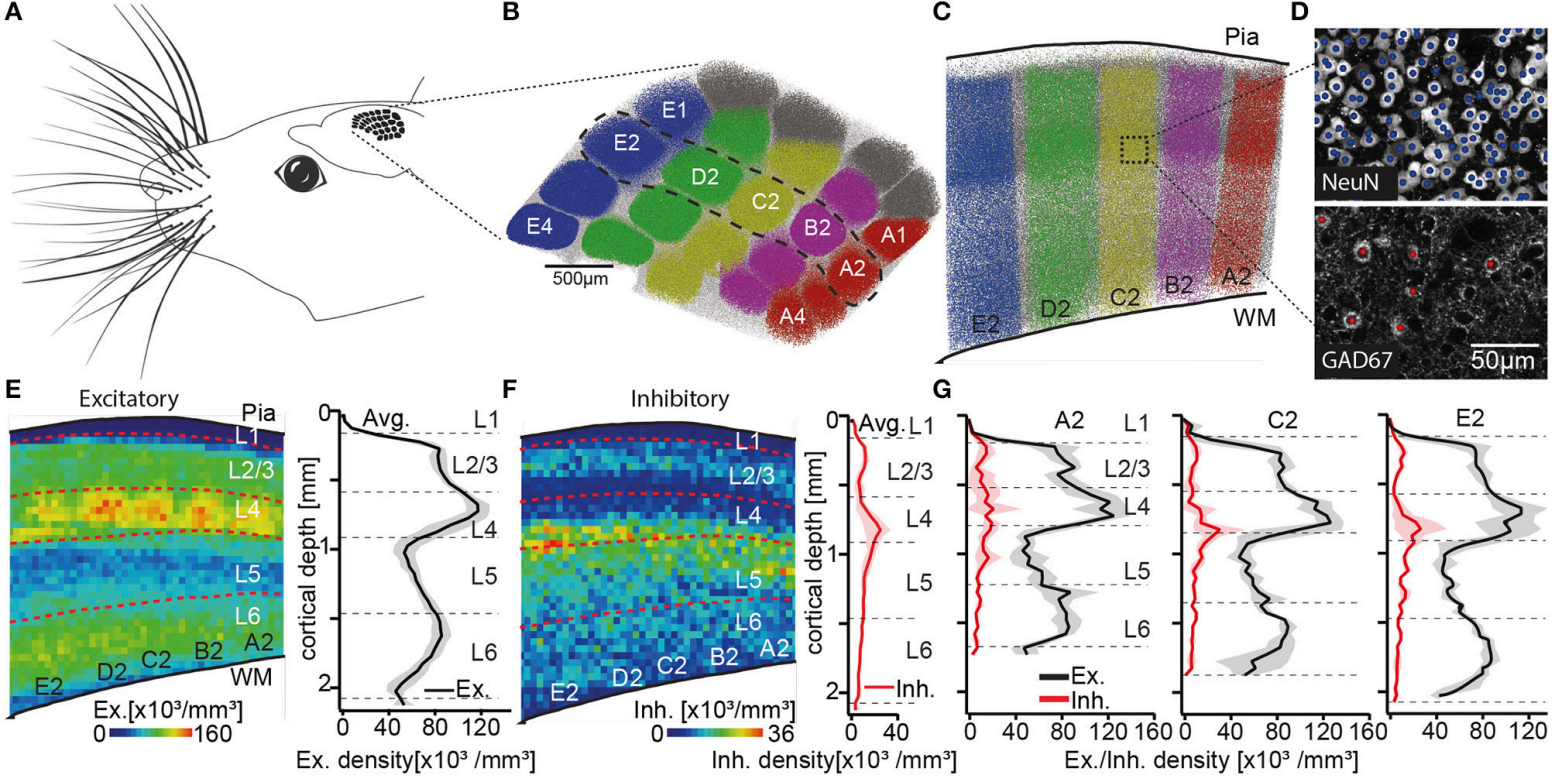
Defining the cortical layers of rat vS1. **(A)** Schematic illustration of the somatotopic organization of vS1 into whisker related barrel columns. **(B)** Top view onto vS1 shows the center locations of all NeuN positive somata with respect to barrel columns. Somata in columns representing whiskers in the A-to-E row (i.e., top-to-bottom of the snout) are colored in red, pink, yellow, green, and blue, respectively. Somata in columns representing the greek whiskers (i.e., α-δ) and those between columns (i.e., septa) are colored in gray and white, respectively. **(C)** Semi-coronal view of cross-section through vS1, showing barrel columns representing arc-2 whiskers (dashed region in **B**). **(D)** Maximum-z projections of image stacks with center locations of all somata (top: NeuN) and the subset of inhibitory somata (bottom: GAD67) for example volume in vS1. **(E)** Left: Average distribution of excitatory somata in 2D projection view. Dashed lines represent layer borders. Right: 1D vertical density profile of excitatory somata averaged across entire vS1. Shaded regions reflect SD. **(F)** Corresponding to panel E: distribution of inhibitory somata (left), 1D vertical density profile of inhibitory somata (right panel). **(G)** 1D vertical density profiles of excitatory and inhibitory somata averaged across columns representing A2, C2, and E2 whiskers, respectively. Note the shift in depth of layer borders across barrel columns. **(B–G)** are adapted and modified from Meyer et al. (2013) with permission.

To define layer borders, we had previously reported the number and 3D distribution of all excitatory and inhibitory somata in entire rat vS1 (Meyer et al., 2013). We had sliced the brains of four animals (28–29 day old, male Wistar rats) into consecutive 50 μm thick vibratome sections, at an angle that is approximately tangential to vS1. The sections were stained with NeuN (Mullen et al., 1992) and GAD67 (Kaufman et al., 1986), to label the somata of all neurons and to discriminate between excitatory and inhibitory somata, respectively. The tangential cutting plane allowed identification of the L4 barrels, which were extrapolated to barrel columns (Figures 1A–D). Thus, we were able to determine the number and vertical distributions of all excitatory and inhibitory somata for each individual barrel column (Figures 1E–G). The resultant soma density profiles along the respective vertical column axes were then used to define and calculate layer borders (see also Meyer et al., 2010), which are represented by the following distances from the pial surface: L1-L2/3: 157 ± 16 μm, L2/3-L4: 575 ± 57 μm, L4-L5: 900 ± 50 μm, L5-L6: 1,411 ± 28 μm; L6-white matter (WM): 1,973 ± 44 μm (Meyer et al., 2013).

Two caveats about these layer border values should, however, be noted. First, it was not possible to define the border between L2 and L3 based on the overall soma density distribution. This is in line with several previous attempts that failed to identify specific anatomical features that distinguish L2 from L3 (e.g., Lund, 1973; Somogyi et al., 1981; Fitzpatrick et al., 1983; Hendry et al., 1987; Lund and Wu, 1997). L2 and L3 are thus often grouped as one layer (i.e., L2/3), even though evidence for functional differences between neurons in L2 and L3 has accumulated from different cortical areas (e.g., Shepherd and Svoboda, 2005; Bureau et al., 2006; Gur and Snodderly, 2008). In our data, the vertical distribution of inhibitory somata was, in general, different compared to the layer-defining distribution of excitatory neuron somata (Figure 1F). Inhibitory somata were densest in upper L2/3 and at the border between L4 and L5 (Meyer et al., 2011). The distribution of inhibitory somata thus provided a quantitative basis for an anatomical separation between L2 and L3. The average border between L2 and L3 was hence calculated as 296 ± 30 μm. Potentially reflecting the different developmental origins of excitatory (Gorski et al., 2002) and inhibitory neurons (Anderson et al., 1997), the different vertical soma distributions question whether grouping inhibitory neurons by their soma locations within cortical layers is an appropriate strategy to describe the organizational principles of inhibitory circuits. For example, the densest distributions of inhibitory neurons in upper L2/3 and at the L4/5 border could underlie the observation that sensory-evoked firing rates of excitatory neurons that are located at these depths are typically much lower than those in deeper parts of L2/3 and L5 (e.g., de Kock et al., 2007). For the remainder of this review article, we will thus restrict our analyses of layer-specific structure and function to excitatory cell types.

The second caveat is that layer borders deviate between barrel columns. The differences do not reflect a linear scaling with cortical thickness. Instead, we found that the diameter of cortical barrel columns compensates for differences in cortical thickness across vS1, resulting in largely the same volume for barrel columns that represent whiskers of the same row along the animals' snout (Egger et al., 2012). More specifically, barrel columns representing whiskers at the bottom of the snout, the so-called E-row, have a volume that is more than three times larger compared to those barrel columns that represent whiskers at the top of the snout (i.e., A-row). The cortical depth changes substantially within and across whisker rows (e.g., by 303 μm from A2 to E2), whereas the location and thickness of L4 is largely preserved across barrel columns (e.g. the depth of L4 changes only by 116 μm from A2 to E2) (Figure 1G). Consequently, the depths and extents of the cortical layers change in a whisker-specific manner. Assigning functional data to a particular cortical layer by measuring the recording or imaging depth, may hence result in mixing neurons from different layers. For the remainder of this review article, we will thus restrict our analyses to the column representing the D2 whisker.

## Defining structure-function cell types in rat vS1

**Figure 2:**
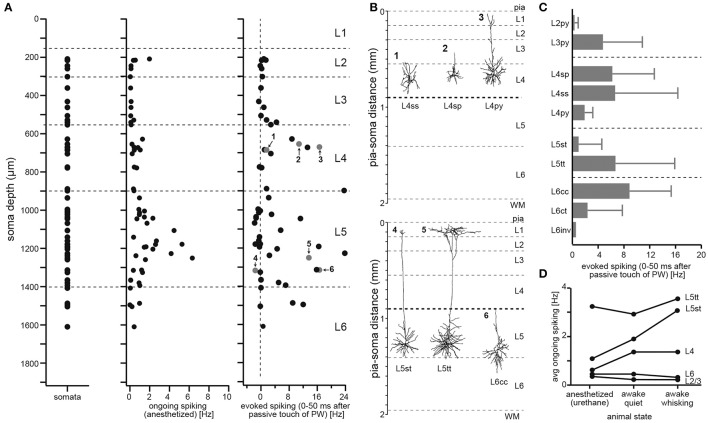
Defining the 10 major excitatory cell types of rat vS1. **(A)** Soma depth locations of *in vivo* recorded/reconstructed excitatory neurons (left). Neuron morphologies were registered to geometrical reference frame of vS1 with ~50 μm precision. Action potentials elicited in anesthetized rats by each of the reconstructed excitatory neurons during periods of ongoing activity (center) and during passive deflection of the somatotopically aligned, so-called principal whisker (PW; right). Evoked spiking rates represent activity after PW stimulation minus ongoing spike rates (panels were modified from (Oberlaender et al., 2012)). **(B)** Example neuron morphologies located at the same depth within L4 (top) and L5 (bottom) illustrate heterogeneity of structural and functional properties within layers. **(C)** Cell type-specific evoked spiking during PW passive deflections. **(D)** Cell type-specific spiking during different behavioral states (adapted and modified from de Kock and Sakmann, 2009 with permission).

To define cell types of excitatory neurons, we had previously reported a dataset that comprised the *in vivo* activity and 3D morphology of individual neurons (Oberlaender et al., 2012), whose somata were located across the entire cortical depth (Figure 2A). We had performed cell-attached recordings in anesthetized animals (28–35 day old, male Wistar rats) and measured spiking patterns during periods of ongoing activity (i.e., without stimulation) and during passive deflections of the somatotopically aligned whisker (de Kock et al., 2007). Following the functional measurements, the recorded neurons were labeled with biocytin (Pinault, 1996; Narayanan et al., 2014), which allowed for *post hoc* reconstructions of their soma, dendrite and intracortical (IC) axon morphologies (Narayanan et al., 2015). The neuron tracings were augmented with reconstructions of the pial surface, WM tract and barrel field in L4, which allowed for precise registration into an average geometrical reference frame of rat vS1 (Egger et al., 2012). Registration compensated for variability in cutting angle and tissue shrinkage across animals, allowing to determine the 3D position of the recorded neuron with ~50 μm accuracy. Combining this dataset with the layer borders described above, we were able to investigate how neuronal morphology and *in vivo* spiking correlate with the neurons' locations in L2-6 (Oberlaender et al., 2012).

In line with several studies that investigated activity patterns across layers—in different sensory systems, species and during different behavioral states (e.g., Vinje and Gallant, 2000; Deweese et al., 2003; Brecht et al., 2004; Olshausen and Field, 2004)—our dataset showed that spiking activity during periods of ongoing activity is sparse (1.09 ± 1.32 Hz, *n* = 57), with neurons in L5 representing, on average, the most active population (1.72 ± 1.56 Hz, *n* = 29). During whisker stimulation, spiking activity increased primarily within L4 and L5 (Figure 2A). However, whisker-evoked (and ongoing) spike rates deviated substantially within each layer, ranging from neurons that did not respond to the stimulus (or decreased spiking compared to ongoing periods) to neurons that increased spike rates by more than 20 Hz within the first 50 ms after stimulation. Both observations, first that sensory stimulation evokes spiking activity most prominently within L4 and L5, and second that responses are highly variable within layers, are generalizable to other stimuli, sensory systems and species (Harris and Shepherd, 2015).

Figure 2B shows the dendrite morphologies of three example neurons that were located approximately at the same cortical depths within L4 and L5, respectively. The neurons differed in their whisker-evoked spike rates and had different dendrite morphologies. We therefore investigated whether the morphological differences of neurons within and across layers can account for the functional variability. We extracted morphological and topological dendrite parameters (see Oberlaender et al., 2012; Narayanan et al., 2015, for a list and definition of the parameters) for 153 *in vivo* recorded/labeled neurons and applied an objective clustering algorithm (Ankerst et al., 1999) to subdivide our sample into dendritic cell types. The classification determined 10 dendritic cell types, which resembled those previously reported in several studies that performed *in vitro* recording/labeling experiments in acute brain slices (see Feldmeyer et al., 2013 for a review). We adopted the cell type naming conventions from the *in vitro* studies and refer to excitatory neurons in vS1 as: L2 pyramidal neurons (L2py), L3py (Petersen and Crochet, 2013), L4py, spiny stellates and star pyramids in L4 (L4ss and L4sp) (Staiger et al., 2004), slender- and thick-tufted pyramids in L5 (L5st and L5tt) (Wise and Jones, 1977), and corticocortical and corticothalamic pyramids in L6 (L6cc and L6ct) (Zhang and Deschenes, 1997; Kumar and Ohana, 2008). The group of L6cc is subdivided into “typical” and “atypical” neurons (DeFelipe and Farinas, 1992), and we refer to the subtypes as L6cc and L6inv, respectively. Typical L6cc are characterized by the lack of apical tuft dendrites (Zhang and Deschenes, 1997; Kumar and Ohana, 2008). The group of L6inv comprises a variety of rare dendritic morphologies, e.g., neurons whose apical dendrite projects toward the white matter (i.e., inverted pyramids).

Grouping the neurons by their respective dendritic cell type could account for some of the functional variability within layers (e.g., whisker-evoked spiking of L4ss/sp vs. L4py: 6.53 vs. 1.90 Hz, or L5st vs. L5tt: 0.97 vs. 6.69 Hz, Figure 2C). The relationship between dendritic cell type and *in vivo* function is likely to extend to other experimental conditions (Figure 2D). For example, in contrast to L5tt, L5st increase spiking activity during the rhythmic back-and-forth movements of whiskers (i.e., whisking), with spike times being correlated to specific whisker positions (i.e., phase) of the whisking cycle (de Kock and Sakmann, 2009).

## Layer-specific organization of excitatory cell types in rat vS1

Previous *in vitro* studies had revealed several relationships between the dendritic cell type and genetic/molecular profiles (e.g., in L5 Groh et al., 2010), intrinsic physiological properties (e.g., in L5 Hattox and Nelson, 2007), local connectivity patterns (e.g., in L5 Brown and Hestrin, 2009) or brain-wide input populations (e.g., in L5 Kim et al., 2015). Not entirely surprising, we found that the cell type-specific structure-function-relationships extend to *in vivo* activity patterns. The ten classes described above may thus represent the major excitatory cell types of rat vS1, and potentially of all sensory cortices (reviewed in Harris and Shepherd, 2015). Therefore, we quantified the degree to which the structural properties of each (dendritic) cell type are organized with respect to the six (somatic) layers of the cortex.

**Figure 3:**
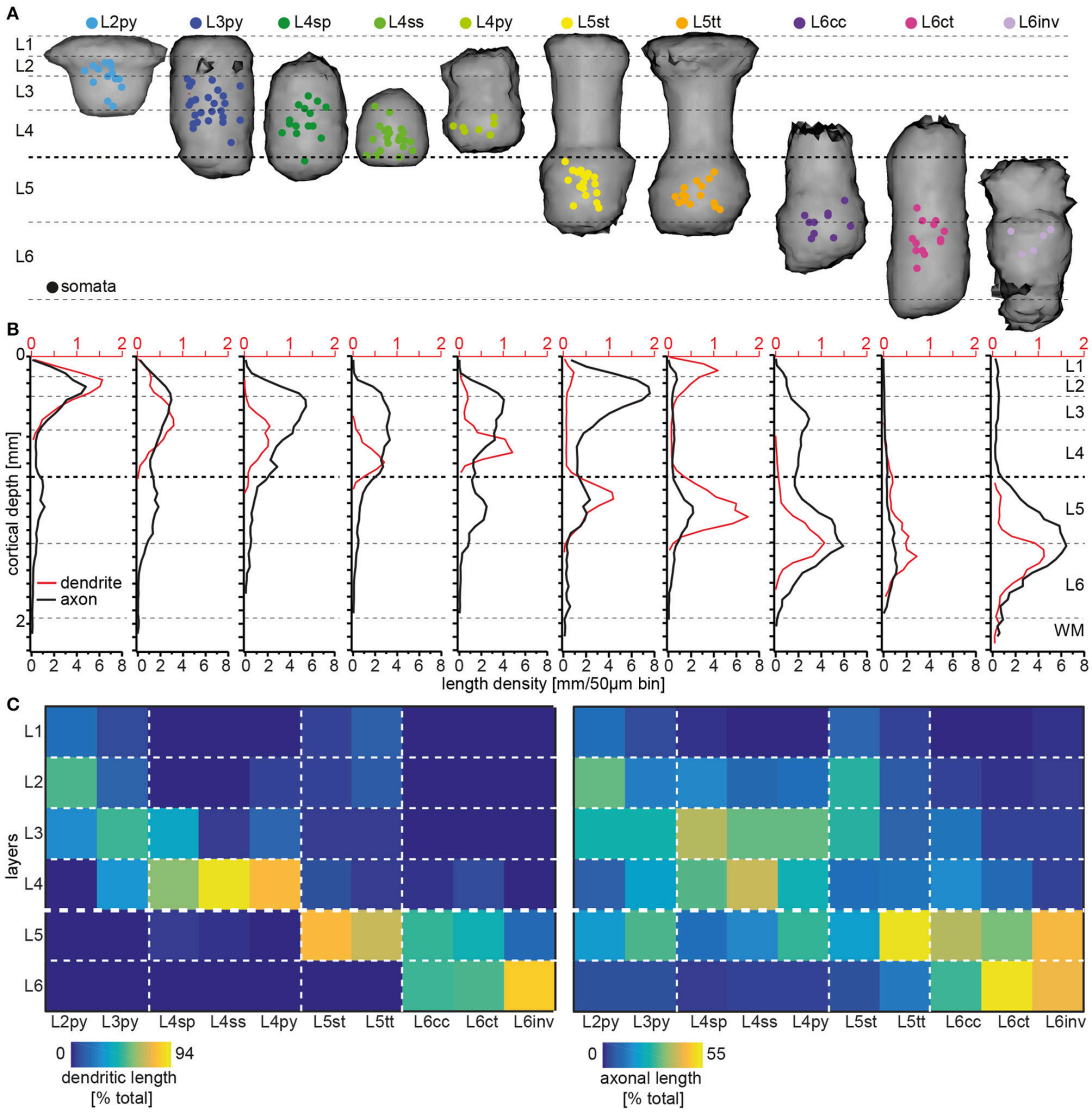
Layer-specific organization of excitatory cell types in rat vS1. **(A)** 3D volumes spanned by registered dendrite morphologies from the 10 major excitatory cell types, respectively. Colored dots denote the soma locations of the *in vivo* recorded/reconstructed neurons (modified from Oberlaender et al., 2012; Narayanan et al., 2015 with permission). **(B)** 1D length density profile of dendrites (red) and axons (black) averaged across all neurons assigned to one of the 10 excitatory cell types (adapted from Narayanan et al., 2015 with permission). **(C)** Quantification of cell type-specific and layer-related dendrite (left panel) and axon (right panel) distributions. The colors denote the relative amount of dendrite/axon per cell type within each layer, normalized to the total dendritic/axonal length of the respective type.

Somata of the respective cell types are not restricted to the layer that is suggested by their naming convention (Figure 3A). Specifically, somata of neurons with the dendritic morphology of L2py are found throughout L2 and L3, but are more frequent within L2 (see Table 1 for the fraction of neurons per cell type per layer). Similarly, somata of L3py are distributed throughout L3 and L4, but are not found in L2. In contrast, somata of L4ss and L4py are largely restricted to L4, whereas those of L4sp are also found in lower L3. L5st and L5tt are restricted to L5, with L5st being more abundant in upper L5 (i.e., L5A: ~80/20% L5st/L5tt) compared to L5tt that are more abundant in deep L5 (i.e., L5B: ~40/60% L5st/L5tt) (Oberlaender et al., 2011). Somata of L6ct and L6inv are largely restricted to L6. In contrast, L6cc are distributed around the L5/6 border (i.e. in deep L5, see also Kasper et al., 1994 and upper L6). Thus, within each layer, somata from multiple excitatory cell types intermingle. The only layer border that separated between somata of different excitatory cell types was the L4/5 border (Narayanan et al., 2015).

**Table 1:** Number of somata, and length of dendrites and axon that each of the 10 major excitatory cell types contributes to each of the six layers of the modeled barrel column.

	**L2py**	**L3py**	**L4sp**	**L4ss**	**L4py**	**L5st**	**L5tt**	**L6cc**	**L6ct**	**L6inv**
**SOMATA**										
L1	34									
L2	1,074	52								
L3	725	1,501	413	113						
L4		1,028	1,024	2,336	517					
L5		67	248	4		1,446	1,106	719	260	12
L6								648	3,711	778
**DENDRITE [m]**										
L1	7.9	2.7				0.9	3.4			
L2	15.4	5.7	0.1		0.5	0.8	2.1			
L3	8.9	20.3	2.2	0.3	1.3	0.7	0.9			
L4	0.1	21.3	5.0	8.1	6.2	2.0	1.5	0.4	2.3	
L5		1.7	0.9	0.3		16.6	21.9	10.4	13.0	2.6
L6						0.1	0.2	8.7	37.2	11.1
**AXON [m]**										
L1	16.1	15.3	2.1	0.3	0.3	14.0	1.1	1.2	0.1	0.7
L2	46.5	45.7	19.4	12.3	8.8	45.0	3.0	5.7	0.7	1.5
L3	38.6	84.0	48.6	43.6	22.6	47.5	3.7	24.7	2.2	2.9
L4	12.2	73.4	43.5	59.5	19.9	22.5	5.5	35.0	9.6	3.8
L5	38.3	117.5	17.2	24.7	26.1	42.9	31.2	90.5	38.0	45.7
L6	6.8	20.3	4.9	3.6	3.3	9.6	12.1	96.0	59.3	67.7

Next, we calculated a convex hull around the dendrites from all cells that had been assigned to a respective cell type (Figure 3A). Remarkably, these dendritic “innervation volumes” showed several relationships with layers (see also Elston et al., 1997, 1999). Specifically, dendrites of L2py are restricted to L1-3 and those of L3py are restricted to L1-4. In contrast, dendrites of L4py and L4sp do not innervate L1 and are restricted to L2-4. Dendrites of L4ss are largely confined to L4. Dendrites of L5st and L5tt extend across L1 to L5 and terminate at the L5/6 border. Dendrites of L6cc and L6ct range from L4-6, whereas those of L6inv remain within L5 and L6. Thus, even though soma distributions of the 10 cell types are only loosely related to layers and the borders between them, and dendrites of each cell type extend across multiple layers, the dendrite distributions display cell type-specific relationships with cytoarchitectonic landmarks (Figure 3B).

Finally, we investigated whether IC axon distributions of each cell type are also organized with respect to layers. In a previous study, we had shown that the IC axon projections of individual excitatory neurons are correlated with their respective dendritic cell type (Narayanan et al., 2015). The cell type-specific axon differences were only partly reflected by parameters such as overall path lengths or topology, but originated primarily from different vertical (i.e., laminar, Figure 3B) and horizontal (i.e., trans-columnar) projection patterns (Narayanan et al., 2015). Specifically, axons of L2py densely innervate L1-3 and less densely L5, with the two innervation peaks coinciding with the centers of L3 and L5, respectively. Axons of L3py have similar axon projection patterns compared to L2py, but innervation of L5 is as dense as innervation of L3, where the peak coincides with the L2/3 border. Axons of the three L4 cell types deviate from most other cell types, as they do not innervate L1. Apart from this difference, the vertical axon profile of L4py resembles the one of the L3py. Axons of L4sp and L4ss are restricted to L2-4. However, axons of L4sp are most dense within L2/3 (the innervation peak coincides with the L2/3 border), whereas axons of L4ss are equally dense in L2/3 and L4. Axons of both, L5st and L5tt, innervate L1-5, but in contrast to L5tt, L5st project densely to L2 and L3 (the innervation peak coincides with the L2/3 border). Axons of L6cc and L6inv innervate the entire cortical depth from the pial surface to the WM tract, whereas L6ct axons are sparse and restricted to L4-6. Axons of L6inv are sparse in L1-4, but dense in L5 and L6 (the innervation peak coincides with the L5/6 border). In contrast, axons of L6cc are most elaborate within L5-6, but also abundant in L3-4, and sparsely innervate L1-2.

We conclude that IC axon morphologies are cell type-specific and layer-related, because the peaks, minima and/or vertical extents of their respective axon density profiles coincide with layer borders and/or centers in a cell type-specific manner. Moreover, we had shown previously that the layer-related distributions of cell type-specific IC axon projection patterns are linked to multiple horizontal (i.e., trans-columnar) organizational principles (Narayanan et al., 2015). With the exception of L4ss and L6ct, the majority of axon from neurons of each cell type is located outside the barrel column containing the soma. These trans-columnar, horizontal patterns are subdivided into three principles: (1) axons that extend widest along barrel columns that represent whiskers of the same row (i.e., “rowish” axons), (2) axons that extend widest orthogonal to the row [i.e., along barrel columns that represent whiskers of the same arc (“arcish”)], or (3) axons that extend unspecifically to all neighboring columns and even beyond (Narayanan et al., 2015). Interestingly, and independent of the cell type, arcish axons are primarily confined to L1-4, rowish axon projections to L5-6.

In summary, dendrite and axon distributions of the 10 excitatory cell types in rat vS1 are organized with respect to cytoarchitectonic layers. However, the laminar landmarks that coincide with the respective dendrite or axon distributions are different and specific for each cell type. For example, even though somata of L2py and L3py intermingle within L3, the vertical extents of their dendrite distributions coincide with the vertical extents of L1-3 and L1-4, respectively. The vertical axon distributions of these two cell types delineate the center and extent of L5, whereas their peak axon densities in the superficial layers coincide with the center of L2 and the L2/3 border, respectively. To provide a comprehensive overview of the degrees to which dendrite and axon distributions of the different cell types are organized with respect to layers, we calculated the relative amount of dendrite and axon path length within each layer for each cell type (Figure 3C).

## Cell type-specific organization of cortical layers in rat vS1

**Figure 4:**
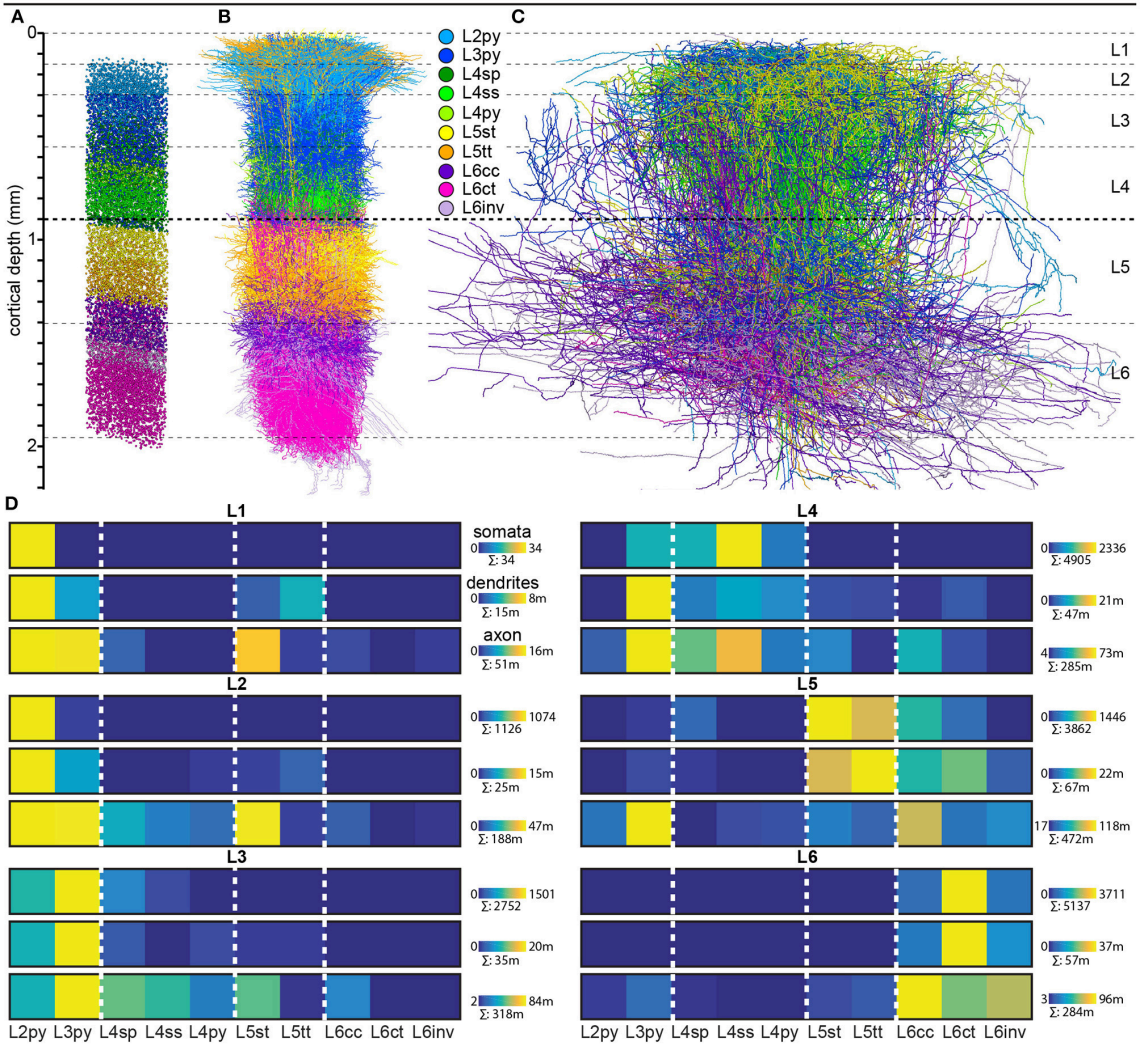
Cell type-specific organization of cortical layers in rat vS1**. (A)** Semi-coronal view of the cell type-specific soma distribution in a dense model of an average cortical barrel column. **(B)** Corresponding to panel A: model of the cell type-specific distribution of dendrites. **(C)** Corresponding to **A**: model of the cell type-specific distribution of axons. **(D)** Quantification of cell type-specific structural composition of each layer with respect to somata (top rows), dendrites (center rows), and axons (bottom rows). Dendritic and axonal length represent all neurons in vS1 whose respective dendrites and/or axons extend into the volume of the modeled barrel column. **(A–C)** were adapted and modified from Oberlaender et al., 2012; Meyer et al., 2013; Egger et al., 2014; Narayanan et al., 2015 with permission.

Finally, we quantified the cell type-specific structural composition of each layer. We had previously reported an approach to generate a dense model of the 3D distributions of cell type-specific somata, dendrites and axons within an average cortical barrel column (Egger et al., 2014). Specifically, we used the 153 reconstructed and registered neuron morphologies to estimate the frequency of occurrence of somata from each cell type along the vertical cortex axis (i.e., at 50 μm resolution), as well as the relative overlaps between cell types (Egger et al., 2014). This allowed assigning each neuron from the measured and registered soma distribution to one of the ten cell types (Figure 4A). The resultant model of an average barrel column representing the D2 whisker comprises 17,816 excitatory somata (see Table 2 for the number of neurons per cell type). Each soma in this model is represented by a dendrite morphology of the respective cell type (Figure 4B), whose registered soma depth was within 50 μm from the location in the model (Egger et al., 2014). This upscaling of the reconstructions resulted in a total dendritic path length that is found within an average barrel column of 246 m (i.e., originating from all excitatory neurons within entire vS1; see also Table 2 for dendritic path lengths contributed by each cell type). Similarly, we upscaled the registered axon morphologies to the respective number of neurons per cell type (Figure 4C). The resultant total (i.e., from all excitatory neurons in vS1) axonal path length within the average barrel column model is 1,599 m (see Table 2 for axon path lengths by each cell type).

**Table 2:** Number of somata, and length of dendrites and axon that each of the 10 major excitatory cell types contributes to the model of an average cortical barrel column.

	**L2py**	**L3py**	**L4sp**	**L4ss**	**L4py**	**L5st**	**L5tt**	**L6cc**	**L6ct**	**L6inv**
Somata	1,833	2,648	1,685	2,453	517	1,446	1,106	1,367	3,971	790
Dendrite [m]	32.2	51.7	8.2	8.7	8.0	21.1	30.0	19.5	52.5	13.7
Axon [m]	158.5	356.3	135.8	144.0	81.0	181.4	56.7	253.0	109.9	122.3

By combining the dense structural model of an average barrel column with our quantification of the layer borders, we estimated the number of somata, as well as the dendritic and axonal path lengths that each of the 10 cell types contributes to each of the six layers (Figure 4D). Within the average column model, the majority of dendrites in L1 originates from L2py, followed by L5tt, L3py, and L5st. The majority of axons originates from L2py, followed by L3py and L5st (see also Table 1). Similar to L1, the majority of the dendrites in L2 originates from L2py, followed by L3py and L5tt. The majority of axons in L2 originates to almost equal amounts from L2py, L3py, and L5st. In L3, L3py, followed by L2py and L4sp contribute the most dendrites, whereas axons in L3 originate from L3py, followed by L5st and the three cell types in L4. The cell type contributing the most dendrites to L4 are L3py, followed by the three L4 cell types. The majority of axon in L4 originates from L3py, L4ss, L4sp, and L6cc. Layer 5 comprises dendrites primarily from L5tt, closely followed by L5st and L6ct. In turn, the majority of axon in L5 originates from L3py, followed by L6cc, followed by similar contributions from all remaining cell types. Layer 6 is more homogeneous than the other layers, with the majority of dendrites and axons originating from the three L6 cell types.

## How cell type-specific are layers and how layer-specific are cell types?

In this review article, we combined several of our previous studies to quantify the degree to which soma, dendrite and axon distributions of the ten major excitatory cell types of rat vS1 are organized with respect to cytoarchitectonic layers. Even though the cell types are purely defined by dendrite morphology, intrinsic properties (Feldmeyer et al., 2013), *in vivo* function (de Kock et al., 2007; Oberlaender et al., 2012), as well as IC axon projection patterns (Narayanan et al., 2015) are cell type-specific. Defining the six layers based on precise measurements of neuron soma distributions revealed that each layer comprises somata of multiple cell types. Nonetheless, dendrite and IC axon innervation patterns of each cell type showed distributions that were related to several laminar landmarks (i.e., center/extent of layers or layer borders). The respective laminar landmarks depended on the cell type, and typically deviated between dendrites and axons within a cell type. Thus, cytoarchitectonic layers can be regarded as a structural reference frame, which gives rise to several cell type-specific dendrite and IC axon projection patterns.

The observation that dendrites and axons of excitatory cortical cell types are organized with respect to laminar landmarks—and may hence be referred to as layer-specific—does not imply that layers are organized in a cell type-specific manner. This is because somata of multiple cell types intermingle within and across layers (i.e., only the L4/5 border represents a cell type border), and the layer-specific organization of dendrites and axons is different for each of those intermingling cell types. Hence, using layers as a synonym for cell types, for example when describing cortical circuits, may yield ambiguous results. For example, the canonical pathway theory suggests that thalamocortical input to L4 is first relayed to L2/3 and then to L5. However, apart from L4ss and L4sp that primarily project their axons to L2-4, L4 comprises also L3py and L4py, which have additional dense axon projections directly to L5. The present quantifications of the layer-related morphological organization of excitatory cell types (Figure 3) and the cell type-specific structural composition of cortical layers (Figure 4) may hence aid future studies to better interpret layer-specific measurements or manipulations with respect to the underlying cell types and circuits.

The layer-related structural organization of excitatory cell types, as reviewed here, is not limited to the local cortical circuitry, but extends to long-range pathways. For example, thalamocortical axons originating in the ventral posterior medial nucleus of the thalamus (VPM) define the extent of L4 and coincide with the L5/6 border in rat vS1. In contrast, axonal projections into vS1 from neurons in the posterior medial nucleus of the thalamus (POm) delineate L1 and coincide with the L4/5 border (Wimmer et al., 2010). In addition to layer-related thalamocortical input patterns, cortical output neurons have specific vertical soma distributions that reflect their respective long-range target areas. For example, VPM-projecting L6ct are located in upper L6 (i.e., L6A), whereas those that project additionally to POm are found in L6B (Zhang and Deschenes, 1997). Similarly, somata of intratelencephalic L5 neurons (ITs)—which are defined by long-range axons that project to the striatum and other cortical areas (Harris and Shepherd, 2015)—are more frequent in L5A, whereas those that project to subcortical targets (i.e., pyramidal tract neurons, PTs Wise and Jones, 1977) are primarily located in L5B. Moreover, we have recently shown that somata of PTs form two sublayers that reflect specific subcortical targets (e.g., POm-projectors are more superficial within L5B than those PTs that innervate the Pons, Rojas-Piloni et al., 2017). The 10 major excitatory cell types of the cortex may thus be divided into further subtypes that differ in their long-range targets, and/or additional structural and functional properties (for a review, e.g., DeFelipe and Farinas, 1992; Spruston, 2008; Harris and Shepherd, 2015) that go beyond those described here (e.g., density and distribution of spines Elston, 2002), and whose respective vertical soma distributions define sublayers within the six cytoarchitectonic layers.

## Author contributions

MO designed the review. RN and DU performed data analysis. All authors wrote the paper.

### Conflict of interest statement

The authors declare that the research was conducted in the absence of any commercial or financial relationships that could be construed as a potential conflict of interest.
